# The Multifaceted Role of STAT3 in Mammary Gland Involution and Breast Cancer

**DOI:** 10.3390/ijms19061695

**Published:** 2018-06-07

**Authors:** Katherine Hughes, Christine J. Watson

**Affiliations:** 1 Department of Veterinary Medicine, University of Cambridge, Madingley Road, Cambridge CB3 0ES, UK; 2 Department of Pathology, University of Cambridge, Tennis Court Road, Cambridge CB2 1QP, UK

**Keywords:** 4T1, breast cancer, chitinase 3-like 1, CLCA, cow, involution, lysosome, mammary gland, microenvironment, STAT3

## Abstract

Since seminal descriptions of signal transducer and activator of transcription 3 (STAT3) as a signal transducer and transcriptional regulator, which is most usually activated by phosphorylation of a specific tyrosine residue, a staggering wealth of research has delineated the key role of this transcription factor as a mediator of mammary gland postlactational regression (involution), and paradoxically, a pro-survival factor in breast cancer and some breast cancer cell lines. STAT3 is a critical regulator of lysosomal-mediated programmed cell death (LM-PCD) during mammary gland involution, where uptake of milk fat globules, and consequent high levels of free fatty acids, cause permeabilisation of lysosomal vesicle membranes, in turn leading to cathepsin protease leakage and cell death. A recent proteomic screen of STAT3-induced changes in lysosomal membrane protein components has highlighted wide-ranging effects of STAT3, which may coordinate LM-PCD via the stimulation of endocytosis, intracellular trafficking, and lysosome biogenesis. In parallel, STAT3 regulates the acute phase response during the first phase of involution, and it contributes to shaping the pro-tumourigenic ‘wound healing’ signature of the gland during the second phase of this process. STAT3 activation during involution is important across species, although some differences exist in the progression of involution in dairy cows. In breast cancer, a number of upstream regulators can lead to STAT3 activation and the effects of phosphorylation of STAT3 are equally wide-ranging. Recent studies have implicated microRNAs in some regulatory pathways. In this review, we will examine the multifaceted role of STAT3 in mammary gland involution and tumourigenesis, incorporating a review of these fundamental processes in tandem with a discussion of recent developments in this field.

## 1. Introduction

**Figure 1:**
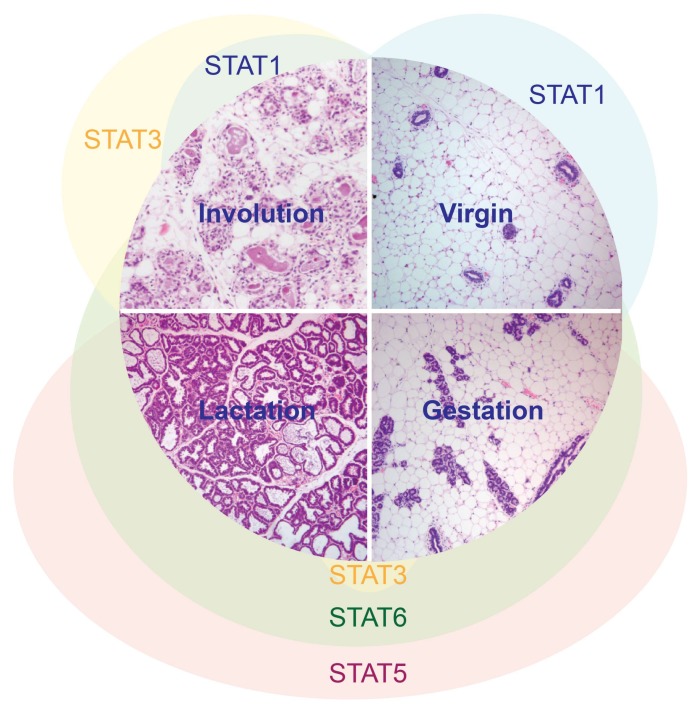
STAT activation patterns during the cycle of postnatal mammary gland development.

STAT3 was first described as an acute phase response regulator in the liver, where its transcriptional activity was activated by phosphorylation of a single tyrosine residue [1,2]. In the subsequent two decades, a plethora of studies has delineated the initially surprising, and key, role of this transcription factor as a mediator of mammary gland postlactational regression (involution) (Figure 1), and paradoxically, as a pro-survival factor in breast cancer. In this review, we will examine the multifaceted role of STAT3 in mammary gland involution and tumourigenesis, incorporating a historic review of these fundamental processes with a discussion of recent developments in this field.

## 2. STAT3 in Mammary Gland Involution

Mammary gland involution comprises the process of glandular postlactational regression that is initiated by weaning. This is widely appreciated to be a dynamic and dramatic process, encompassing two primary waves of cell death and the marked remodelling of the stromal compartment. While most research has been carried out using genetic models in the mouse, such changes have recently been illustrated in the human breast by diffusion tensor magnetic resonance in the first year following the cessation of lactation. As observed in the mouse, the size and fibroglandular fraction of the human breast in the postweaning period decreased significantly when compared to lactation [3].

The process of involution is both exquisitely complicated and highly controlled, with STAT3 being a key player in this process [4,5,6]. One striking element of the progression of involution in mice is that it comprises two distinct and well-characterised stages [7,8]. Microarray data has subsequently enabled the further division of the two phases into multiple steps [9].

### 2.1. STAT3 as a Regulator of Cell Death during Involution

Experiments using mice that have a unilaterally sealed inguinal mammary gland teat have demonstrated that removal of the suckling stimulus, and the associated milk stasis, lead to phosphorylation of STAT3 and the commencement of involution [10]. Thus, the initial upregulation of pSTAT3 at the onset of involution is not due to a systemic decrease in circulating lactogenic hormones. Data derived from examination of leukemia inhibitory factor (LIF) deficient mice show that LIF is the initial activator of STAT3 during involution [11]. Whilst LIF is almost undetectable in the mammary gland during lactation, high levels of expression are seen for the first three days of murine postlactational regression. Further evidence supporting the importance of LIF as an upstream regulator of STAT3 at this time has been derived from experiments in which the implantation of LIF containing pellets during lactation increased mammary epithelial cell death [12]. 

Teat sealing experiments have also demonstrated that transforming growth factor beta 3 (TGF-β3) expression is induced by milk accumulation. STAT3 activity is suggested to be regulated by TGF-β3 expression, which thus impacts cell death during the first phase of involution [13].

Interestingly, mammary epithelial CCAAT/enhancer binding protein delta (*C/ebpδ*) mRNA content is low during pregnancy and lactation, but it also exhibits a dramatic, exceeding 100-fold, increase within 12 h of the onset of postlactational regression, or in teat sealed glands [14,15]. When microarray data has been used to categorise the progression of involution into a series of steps, *C/ebpδ* is grouped with genes showing their strongest expression on the first day following the induction of involution [16]. As *C/ebpδ* is a STAT3 target gene, it has been inferred to be an important mediator of the ensuing cell death in the mammary epithelial cells [17]. The relationship between STAT3 signalling and *C/ebpδ* transcription has been further confirmed by microarray analysis of conditionally induced STAT3 genes in a mammary epithelial cell line (KIM-2), which indicates that STAT3 induces *C/ebpδ* expression, along with a number of other genes, including the negative feedback regulator suppressor of cytokine signaling 3 (*Socs3*) [18].

During the first, reversible and proteinase independent, phase of involution, STAT3 initiates dramatic mammary epithelial cell death [19,20,21,22]. STAT3 deletion results in early embryonic lethality [23] and therefore investigation of the role of STAT3 in mammary epithelial cell death necessitated the development of a murine mammary conditional deletion of *Stat3*. This was achieved through the utilization of the Cre-lox recombination system where a mammary-specific promoter [24], such as whey acidic protein (*Wap*-Cre) [20] or β-lactoglobulin (*Blg*-Cre) [19], controlled the expression of Cre recombinase. Early experiments using these systems demonstrated that conditional deletion of STAT3 from mammary epithelial cells resulted in a profound delay in the progression of involution [19,20].

Intriguingly, it has been demonstrated that involution is unaffected by the deletion of the executioner caspases 3, 6, or 7 or by the overexpression of a viral apoptosis inhibitor, p35 [21]. This observation, taken together with the recognition that expression of the lysosomal cathepsin protease inhibitor Spi2a (serpina3g) drops precipitously, in a STAT3-dependent manner, within 12 h of involution, led to the conclusion that STAT3-regulated cell death during the reversible phase of involution is not achieved via apoptosis, but rather via activation of a lysosomal-mediated programmed cell death (LM-PCD) pathway. During involution, mammary epithelial lysosomes undergo lysosomal membrane permeabilization and STAT3 upregulates the expression of cathepsins B and L [21,25]. Involution is retarded in mice deficient in cathepsin L, and reduced cytosolic levels and activity of this cysteine protease are also observed in mice with deleted p55α and p50α regulatory subunits of phosphatidylinositol 3-kinase (PI3K), which also exhibit delayed involution [26].

The trigger for STAT3-regulated LM-PCD in mouse mammary epithelial cells is the uptake of milk fat globules, which become toxic to the cell as they are delivered to lysosomes. High levels of free fatty acids cause permeabilisation of lysosomal vesicle membranes in mammary epithelial cells in vitro, which in turn leads to cathepsin leakage and cell death [22]. A recent proteomic screen of STAT3-induced changes in lysosomal membrane protein components has highlighted that STAT3 has wide-ranging effects and may stimulate endocytosis, intracellular trafficking, and lysosome biogenesis and localisation within mammary epithelial cells to coordinate LM-PCD [25].

Proteinases, including the matrix metalloproteinases (MMPs) MMP2 (gelatinase A), MMP3 (stromelysin 1), and MMP9, together with the serine proteinase urokinase-type plasminogen activator [7,27] degrade the mammary basement membrane irreversibly during the second stage of involution. This phase is classically considered to commence at 48 h post weaning in the mouse [4], and the early teat sealing experiments already described demonstrated that it does not occur in sealed glands in mice in which the contralateral gland is left open [10]. Similarly, systemic factors, including endogenous release of glucocorticoids or exogenous administration of hydrocortisone, can impede the progression of the second phase of postlactational regression [7].

LIF-induced STAT3 activity causes the upregulation of oncostatin M (OSM) and its receptor from approximately 48 h of involution and OSM is considered to be the primary cytokine activating STAT3 during the second phase of involution, with mice that are deficient in the OSM receptor exhibiting a delayed involution phenotype [28]. OSM has been demonstrated to promote expression of MMP3, MMP12, and MMP14 [28].

TGF-β has also been suggested to have a role in the regulation of cell death during the second phase of involution, with mice with a mammary epithelial-specific deletion of exon 2 of the TGF-β receptor 2 (*Tgfbr2*) gene exhibiting clearly detectable alveolar structures containing milk at one week of involution. Interestingly, more abundant activated STAT3 was detected at day 3 of involution in these mice, which the investigators attributed to local alveolar distension by milk [29].

### 2.2. STAT3 as a Modulator of the Involution Inflammatory Microenvironment

A panel of genes that are associated with the acute phase response and innate immunity are dramatically upregulated at the onset of involution, including serum amyloid A1 and A2 [16,30]. Of these genes, a subgroup, including orosomucoids 1 and 2, secretory leukocyte protease inhibitor, and leucine-rich α2-glycoprotein 1, is regulated in a STAT3-dependent manner [27].

**Figure 2:**
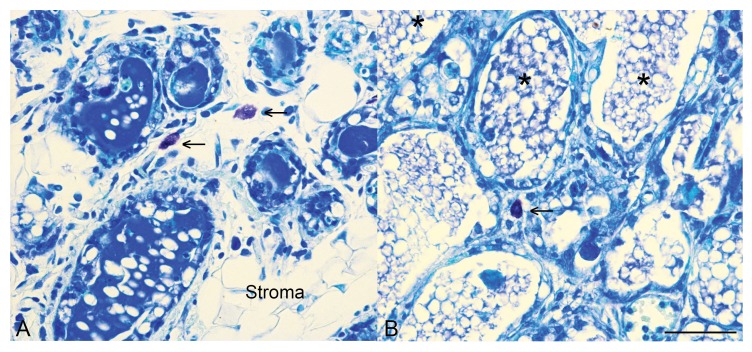
Mast cells are present in the mammary gland during involution. Toluidine blue staining for mast cells (arrows) in control mammary tissue (**A**) and mammary tissue with an epithelial-specific deletion of *Stat3* (**B**) at 96 h of involution. Note also that in control tissue by 96 h of involution there is distinct re-emergence of the stromal compartment, as indicated. In mammary tissue with an epithelial-specific deletion of *Stat3*, more numerous alveoli are retained, many contain intraluminal residual milk (*) and we have demonstrated that at 72 h of involution, mast cell influx is diminished [27]. Scale bar indicates 50 microns.

By contrast, the phenotypic signature of the second phase of mammary involution has been likened to that of a healing wound [16,31,32], with a number of factors suggested to contribute to this immunomodulatory phenotype (Table 1 and Figure 2). We have demonstrated that the second phase involution ‘wound healing’ phenotype is modulated by STAT3, with reduced expression of arginase-1 and Ym1, markers that are associated with immunomodulatory macrophages, and a reduction in mast cell numbers in STAT3-deleted glands [27]. Connective tissue-type mast cells have been suggested to be a source of the plasminogen activator plasma kallikrein during the involution process [33]. Importantly, we have also demonstrated that, in the irreversible phase of involution, STAT3 regulates the expression of chitinase 3-like 1 (BRP-39; human homologue: YKL-40) with abrogation of chitinase 3-like 1 expression in Stat3-deleted glands, and reduced levels of expression in mice deficient in the OSM receptor [27]. Chitinase 3-like 1 is a secretory glycoprotein that is expressed during murine mammary gland involution [16,34], which has been demonstrated to increase MMP9 expression and cell invasiveness when overexpressed in vitro in mammary epithelial cells [35]. Chitinase-like proteins are associated with tissue remodelling and the regulation of innate immune pathways in a range of contexts [36], and it seems likely that we do not yet appreciate their full importance in mammary gland involution.

Members of the chloride channel regulators, calcium activated (CLCA) family, of proteins have been suggested to possess diverse roles in control of cellular proliferation, differentiation of epithelial cells, transmembrane trafficking of anions, tumour suppression, and the activation of macrophages [43,44,45,46]. Murine family members *mClca1*, *mClca2*, and *mClca5* (homologous to human *CLCA2*; *hCLCA2*) are expressed in the mammary gland [43,44,47]. Using mice with a mammary epithelial-specific deletion of *Stat3*, we have demonstrated that STAT3 positively regulates expression of *mClca1* and *mClca2*. Data from KIM-2 cells stimulated with OSM, and from mice deficient in STAT3, indicate that STAT3 negatively regulates *mClca5*. The significance of the relationship between STAT3 and CLCA family members within the mammary gland requires further interrogation, but it is tempting to speculate that their role may encompass epithelial and/or microenvironmental modulation during this phase of dramatic remodeling [47].

### 2.3. The Mammary Epithelial Cell: A Key Player in the Mammary Microenvironment

Mammary epithelial cells are important contributors to the microenvironment of the mammary gland during involution. During murine postlactational regression, CD14 expression by mammary epithelial cells is strikingly upregulated in a STAT3-dependent manner [16,27]. Taken together with other data described below, this suggests that mammary epithelial cells acquire phagocytic properties. Efferocytosis is the phagocytic removal, by neighbouring cells or professional phagocytes, of unneeded cells or those that are exhibiting defects [48]. During involution, mammary epithelial cells are important in the execution of efferocytosis to remove dead cells [39,40,49] and evidence suggests that TGF-β3 may also have a role in this process [50]. It is noteworthy that efferocytosis is thought to contribute to the pro-tumourigenic ‘wound healing’ microenvironment of mammary gland involution [51,52]. As already described, during the early stages of postlactational regression, mammary epithelial cells also utilise phagocytosis in the STAT3-dependent uptake of butyrophilin 1A1-coated milk fat globules amassing in the lumen of mammary alveoli [22].

### 2.4. STAT3 in Dairy Cow Mammary Gland Involution

Mammary gland involution in the dairy cow frequently differs from murine models in that cows in dairy production systems are likely to be in the final trimester of pregnancy during the period of mammary gland involution (termed the dry period) [53], and thus may exhibit what we have described as a ‘parallel pregnancy signature’ [54]. Bovine mammary involution is considered to encompass both a phase of cell death, but also an important period of epithelial cell renewal prior to the next lactation [55,56,57]. Interestingly, when involution is experimentally induced by cessation of milking in non-pregnant dairy cows that are close to the point of peak lactation in mid-lactation, increased phosphorylation of STAT3 is observed by 72 h [58,59]. As would be anticipated, the dynamics of the involution process in cows are strikingly different from that of rodents, and, following abrupt cessation of milking at mid lactation, milking can be reinitiated after seven days of non-milking, with a 91% milk yield recovery and milk composition similar to that observed prior to cessation of lactation [60].

## 3. STAT3 in Breast Cancer

STAT3 was first described to be constitutively active in invasive, but not benign, breast tumour biopsies [61]. In a subsequent study, 57% of analysed breast tumour samples exhibited moderate to high levels of nuclear pSTAT3 expression [62]. These results were later echoed by those of another group, reporting that 68% of the breast tumours analysed exhibited nuclear pSTAT3, and the enrichment of STAT3 signature genes, considered to potentially represent ‘critical effectors of STAT3 activation in malignancy’ [63]. It is thus, apparently, a striking paradox that STAT3 regulates cell death during mammary gland involution, and yet promotes survival of breast cancer cells [64,65].

STAT3 signalling in breast cancer cells may confer a survival advantage by promoting aberrant expression of oncogenic target genes [66]. For example, *Bcl-xL*, *MCL1*, and *survivin* are STAT3 target genes [67] that are downregulated in breast cancer cell lines treated with the small molecule hydroxamic acid–based and benzoic acid–based STAT3 inhibitors [68]. STAT3 has also been shown to upregulate the expression of the lysosomal enzyme cathepsin B that has been implicated in mammary tumourigenesis [69].

In breast cancer, as in physiological contexts, STAT3 can be activated by cytokine receptors, particularly receptors for interleukin-6 (IL-6) family cytokines, including the OSM receptor, as described in the second phase of involution. However, it is important to note that STAT3 activation in various cancers may also be achieved by a number of other pathways, including downstream of receptor tyrosine kinases (particularly epidermal growth factor receptor [EGFR]), non-receptor tyrosine kinases, some serine kinases, G protein coupled receptors, Rho GTPases, cadherin engagement, and toll-like receptors [70].

MicroRNAs (miRNAs) also fulfil a crucial role in regulating STAT3 signalling [70]. miR-519d directly targets *STAT3* for downregulation, thus functioning as a tumour suppressor in breast cancer. Consequently, breast cancer tissues with a low miR-519d expression have higher levels of STAT3 protein. miR-519d is often downregulated in breast cancer [71]. Members of the let-7 miRNA family are also widely accepted to be tumour suppressors that indirectly regulate STAT3 [70]. However, STAT3 can also repress let-7 via transcription of *Lin*-28, resulting in the upregulation of the let-7 target, high-mobility group A protein 2 (HMGA2). Interestingly, HMGA2 promotes epithelial-to-mesenchymal transition that is driven by OSM [72]. An investigation of miRNAs expressed throughout a 16 time point mouse mammary developmental cycle (including involution) showed that they were expressed in clusters, which were proposed to be co-regulated, and that these clusters contained breast cancer-associated miRNAs that were significantly enriched [73].

We have previously reviewed the role of STAT3 and the inflammation/acute phase response in involution and breast cancer [74]. More recently, we have shown that in the absence of STAT3, involution is associated with an impairment of the acute phase response and a reduction in both the number of infiltrating immune cells and the phenotype of stromal macrophages [27]. Other work has demonstrated that, once activated, STAT3 can modulate the tumour microenvironment, either directly or indirectly, by a plethora of means [66]. For example, in MDA-MB-231 breast cancer cells, STAT3 interacts with hypoxia-inducible factor 1 α (HIF1α) protein to activate HIF target genes, including vascular endothelial growth factor [75]. Other investigators have demonstrated that a constitutively active mutant form of STAT3 expressed in immortalized human mammary epithelial cells induces expression of MMP9 mRNA and protein and furthermore that MMP9 expression correlates with nuclear pSTAT3 expression in breast cancer tissues [62]. In a mouse model of activated *ErbB2*-driven breast cancer, it was found that STAT3 does not have a role to play in tumour initiation, but it has a dramatic effect on metastatic progression with a 12-fold reduction in the number of metastatic lesions in the lungs of animals with STAT3-null/*ErbB2* tumours when compared to STAT3-wild-type/*ErbB2* tumours. The authors concluded that this was a consequence of a reduction in both angiogenic and inflammatory responses that were mediated by C/ebpδ [76]. Similarly, mice harbouring a constitutively active STAT3 allele (STAT3C) in a transgenic MMTV*-Neu (ErbB2)* background, exhibited more invasive mammary tumours with earlier onset than those with wild-type STAT3 alleles [77]. More recently, a role for STAT3, which was expressed in the epithelium, in promoting an immunosuppressive tumour microenvironment during the early stages of tumour initiation and progression has been demonstrated in a polyomavirus middle T mouse model [78].

STAT3 and STAT5 may be activated in tandem in breast cancer, with 29% of tumours exhibiting this characteristic. Such tumours are described as being more differentiated than those in which STAT3 alone is activated [79]. In breast cancer, the relationship between different STAT family members and their receptor-associated Janus kinases (JAKs) is complicated. Persistent prolactin receptor signalling has been shown in STAT1-deficient mammary epithelial cells, and this results in the activation of JAK2, STAT3 and STAT5A/5B, and the subsequent development of oestrogen receptor-α-positive mammary tumours [80,81]. It therefore appears that the phosphorylation of STAT3 and STAT5 requires JAK2, and that the perpetual stimulation of the prolactin receptor-JAK2-STAT3/5A/5B axis constitutes a survival signal for neoplastic mammary epithelial cells [81,82].

## 4. STAT3 Expression in 4T1 Murine Mammary Carcinoma Cells

Given the preceding discussion regarding the importance of STAT3 signalling in breast cancer, it is perhaps unsurprising that we and others have observed robust levels of expression of pSTAT3 in tumours resulting from implantation, into the mammary fat pad, of the syngeneic murine mammary carcinoma cell line 4T1 [47,83]. We observed the highest levels of pSTAT3 immunoreactivity at the invasive edge of the tumours where pSTAT3 was not restricted to the neoplastic cells alone, with positive nuclear pSTAT3 immunoreactivity being observed in cells with morphology that is consistent with tumour cells, together with fibroblasts and immune cells [47]. This finding illustrates the importance of STAT3 activity in the tumour microenvironment, as well as tumour cells themselves.

Using the 4T1 model other investigators have demonstrated the importance of myeloid derived suppressor cells (MDSCs). When compared to the mouse mammary carcinoma cell line EMT6, 4T1 cells exhibit higher levels of IL-6 expression in culture, and in a series of experiments using these two cell lines, it has been demonstrated that IL-6 expression positively impacts the recruitment of MDSCs and metastatic potential. The authors attribute the augmentation of STAT3 activity in the tumour cells to the secretion of IL-6 and soluble IL-6Rα by the MDSCs [84].

Interestingly, administration of the natural napthoquinone compound shikonin has been demonstrated to reduce the in vivo growth of tumours that are derived from implantation of 4T1 cells, and this is suggested to be through modulation of STAT3 and Oct3/4 expression [83]. Nifuroxazide, an antidiarrhoeal agent, also inhibits STAT3 in 4T1 cells. In vitro administration of nifuroxazide to 4T1 cells reduces pSTAT3 levels, together with the expression of MMP2 and 9, and cell motility is reduced. The same authors demonstrated that, in vivo, nifuroxazide administration reduced the growth and metastasis of 4T1 cell-derived tumours [85]. Thus, it can be inferred that STAT3 activity in 4T1 cells, and potentially also within the tumour microenvironment, is likely critical to the invasive and metastatic phenotype of 4T1 cells.

## 5. STAT3 in Mammary Cancers in other Species

Whilst mammary neoplasia is not a disease that is limited to humans, relatively sparse data currently exist regarding the importance of STAT3 activity in mammary tumours in animals, particularly when considering studies correlating pSTAT3 expression directly with clinical outcome. pSTAT3 levels have been shown to be significantly higher in metastatic canine mammary tumours than in non-metastatic examples, and have similarly been demonstrated to be higher in canine mammary carcinoma cell lines that were derived from tumours with metastatic potential [86,87]. Feline mammary tumours are frequently aggressive, have been suggested to be suitable for use as a spontaneous model of human disease, and have attracted widespread attention for the evaluation of potential prognostic markers [88,89]. Assessment of feline mammary tumours has demonstrated that nuclear pSTAT3 positively correlates with increasing histological grade, decreasing formation of tubules, and increasing replicative activity [90]. STAT3 phosphorylated on serine 727 has also been evaluated, with similar correlations noted, including a positive relationship between levels and increasing pleomorphism, cellular proliferation, and histological grade [91]. Finally, we have demonstrated that a subset of equine mammary carcinomas, which are rare but frequently aggressive tumours, exhibit nuclear localization of STAT3 [92].

## 6. STAT3 Inhibition

Given its undisputed status as a tantalizing target for inhibition, it is unsurprising that considerable focus is placed upon the development of STAT3 inhibitors. A full discussion of the development of such inhibitors is beyond the scope of this discussion, and this topic has recently been reviewed elsewhere [65,93]. It is also important to consider that STAT3 activating cytokines, such as IL-6 [94], or OSM [95], may also represent potential therapeutic targets. Another potential target for inhibition of STAT3 signalling is the upstream activating kinase, JAK2. The well-established JAK2 inhibitor ruxolitinib is currently in phase II clinical trials for patients with metastatic triple negative breast cancer [96].

## 7. Conclusions

The plethora of studies documenting the importance of STAT3 in mammary gland involution and breast cancer underlines the critical importance of this transcription factor in the regulation of physiological cell death during postlactational regression, and paradoxically, in providing a survival advantage for neoplastic cells in breast cancer. In this context, the role of STAT3 in the tumour microenvironment is critically important, and this, coupled with the fact that STAT3 is not normally activated in undifferentiated mammary epithelial cells, may go part of the way to explain this paradox. It is clear that STAT3 also contributes to shaping the immunomodulatory, and pro-tumourigenic, ‘wound healing’ signature of the mammary gland during involution. The critical importance of STAT3 highlights it as a promising target for therapeutic interventions, and although the development of inhibitors of STAT3 is proving to be challenging, it is likely that the field will continue to develop with advances in this area.

## Figures and Tables

**Table 1:** Factors implicated in the ‘wound healing’ signature [16] of second phase mammary gland involution.

Factor	Selected References
**Structural/anatomical factors:**	
Deposition of fibrillar collagen	[31,37]
Lymphangiogenesis	[38]
**Enzymatic factors:**	
High levels of COX-2 expression	[38]
**Cellular factors:**	
Mammary epithelial cell efferocytosis	[39,40]
Alternatively activated or immunosuppressive (IL-10^+^) macrophages	[27,31,32,41]
Foxp3^+^ regulatory T cells	[41]
Mast cells	[27,33,42]

## Abbreviations

C/EBPδ	CCAAT/enhancer binding protein delta
CLCA	Chloride channel regulators, calcium activated
LIF	Leukemia inhibitory factor
MMP	Matrix metalloproteinase
STAT	Signal transducer and activator of transcription
